# The Role of Age, Education, and Digital Health Literacy in the Usability of Internet-Based Cognitive Behavioral Therapy for Chronic Pain: Mixed Methods Study

**DOI:** 10.2196/12883

**Published:** 2019-11-21

**Authors:** Rosalie van der Vaart, Dorine van Driel, Kristel Pronk, Suzan Paulussen, Selma te Boekhorst, Judith G M Rosmalen, Andrea W M Evers

**Affiliations:** 1 Leiden University Faculty of Social and Behavioural Sciences Health, Medical and Neuropsychology Unit Leiden Netherlands; 2 SeniorBeter Practice of Old Age Psychiatry Gendt Netherlands; 3 Dimence Almelo Netherlands; 4 University of Groningen University Medical Center Groningen Department of Psychiatry Groningen Netherlands; 5 University of Groningen University Medical Center Groningen Department of Internal Medicine Groningen Netherlands

**Keywords:** internet-based cognitive behavior therapy, chronic pain, usability, digital health literacy skills, eHealth literacy

## Abstract

**Background:**

Internet-based cognitive behavior therapy (iCBT) can be effective in mental and somatic health care. Research on the feasibility of internet interventions in clinical practice is, however, still scarce. Studies with a focus on the patient regarding usability of interventions and digital health literacy skills are especially lacking.

**Objective:**

The goal of this study was to assess the usability of an iCBT for chronic pain, Master Your Pain, and the relationship between its usability outcomes and the factors age, educational level, and digital health literacy skills. The aims were to determine what changes were needed in the program for sufficient usability and which individual characteristics were related to the usability of the program.

**Methods:**

Patients were recruited from two mental health care practices. A mixed methods approach was used in this study. A qualitative observational study comprising performance tasks in the iCBT program was used to test usability. A quantitative questionnaire was used to measure possible related constructs. Usability was operationalized as the number of tasks that could be completed and the type and number of problems that occurred while doing so. Performance tasks were set up to measure 6 digital skills: (1) operating the computer and internet browser, (2) navigation and orientation, (3) using search strategies, (4) evaluating relevance of content, (5) adding personal content, and (6) protecting and respecting privacy. Participants were asked to think aloud while performing the tasks, and screen activities and webcam recordings were captured. The qualitative observational data was coded using inductive analysis by two independent researchers. Correlational analyses were performed to test how usability relates to sociodemographics and digital health literacy.

**Results:**

A total of 32 patients participated, with a mean age of 49.9 years and 84% (27/32) being female. All performance tasks except one (fill in a diary registration) could be completed independently by more than 50% of the participants. On operational, navigation, and search levels, participants struggled most with logging in, logging out, and finding specific parts of the intervention. Half of the sample experienced problems evaluating the relevance and adding content to the program to some extent. Usability correlated moderately negatively with age and moderately positively with digital health literacy skills but not with educational level.

**Conclusions:**

The results provide insight into what is essential for proper usability regarding the design of an iCBT program considering variations in age, educational level, and digital health literacy. Furthermore, the results provide insight into what type of support is needed by patients to properly use the intervention. Tailoring support among the needs of certain age groups or skill levels could be beneficial and could range from no extra support (only online feedback, as intended) to practical support (an additional usability introduction session) to blended care (combined face-to-face sessions throughout the therapy).

## Introduction

Internet interventions are effective for treating patients with a broad range of mental health problems and supporting patients in their coping and self-management of chronic somatic conditions [1,2]. In general, these types of interventions are therapist-guided, Web-based programs. Patients log in using a personal account and enter a personal dashboard, from which they can follow modules consisting of psychoeducational texts, assignments, relaxation exercises, and diary registrations. Through the program they can also communicate with their care provider, commonly via secured email. In clinical trials, where participants are generally rather homogeneous, these programs have been found to be effective in reaching behavior change. However, less studied is the extent to which these interventions are feasible to use in clinical practice for a heterogeneous patient population [3]. This regards their fit in regular care processes and usability among individual patients and care providers. Over the past few years it has become clear that implementing internet interventions into regular health care is challenging [4-6], but the focus on the needs and skills of patients in this matter has been minimal.

Up until now, little has been known about for which (type of) patients internet interventions would be suitable [7]. A well-established precondition for effective and efficient use of internet interventions is that the programs are easy and intuitive to use for patients [8]. Furthermore, failure to meet the needs and skills of intended users can play a key role in disappointing use [9,10]. The need for assistance while using an internet intervention may imply that the usability of the application must be improved, which could be achieved by redesigning the program to simplify the user interface or offering guidance within the program. Nevertheless, in addition to the usability of an internet intervention being relevant to reach optimal effect, the skill level of the individual using the intervention must be adequate. These usage skills are called eHealth literacy, or digital health literacy, defined as the skills needed to “seek, find, understand, and appraise health information from electronic sources and apply the knowledge gained to addressing or solving a health problem” [11]. An in-depth examination of digital health literacy disclosed six types of health-related internet skills needed when using online health information and interactive interventions: (1) operating the computer and internet browser, (2) navigating and orienting, (3) using search strategies, (4) evaluating relevance and reliability of Web content, (5) adding personal content to the Web, and (6) protecting and respecting privacy [12].

Research on the use of health-related digital applications has shown that sociodemographics, age, and educational level are associated with the skills required to successfully use these applications [13]. Van Deursen et al [13] used performance-based assignments to measure usage skills of people in the general population, which showed that older age and lower educational level were predictive of lower operational and formal skills, the practical skills needed to use and navigate on a computer and on the internet. Additionally, educational level was predictive of informational and strategic skills, indicating that individuals with lower educational levels have more difficulties finding and critically appraising online information [13]. This suggests that elderly and less educated individuals might be more vulnerable groups when it comes to using internet interventions. These users might need more guidance or assistance in using an internet intervention, or they might benefit more from blended care, in which they also have regular face-to-face contact with their therapist [7].

In this study, the usability of an internet-based cognitive behavioral therapy (iCBT) called Master Your Pain was assessed in patients with chronic pain to best facilitate patient needs and skills in using the program. First, we measured to what extent potential users were able to perform relevant assignments related to natural use of the intervention. This was operationalized by assessing the type and number of encountered problems. Second, since the population of chronic pain patients tends to be older and experience with the internet might be less, the associations between age, educational level, digital health literacy skills, and the performance and the number of occurred problems in the iCBT program were explored. This provided preliminary insight into the feasibility of using the intervention in clinical practice and the possible need for tailoring (ie, providing additional assistance or guidance to people working with the program).

## Methods

### Design

**Figure 1:**
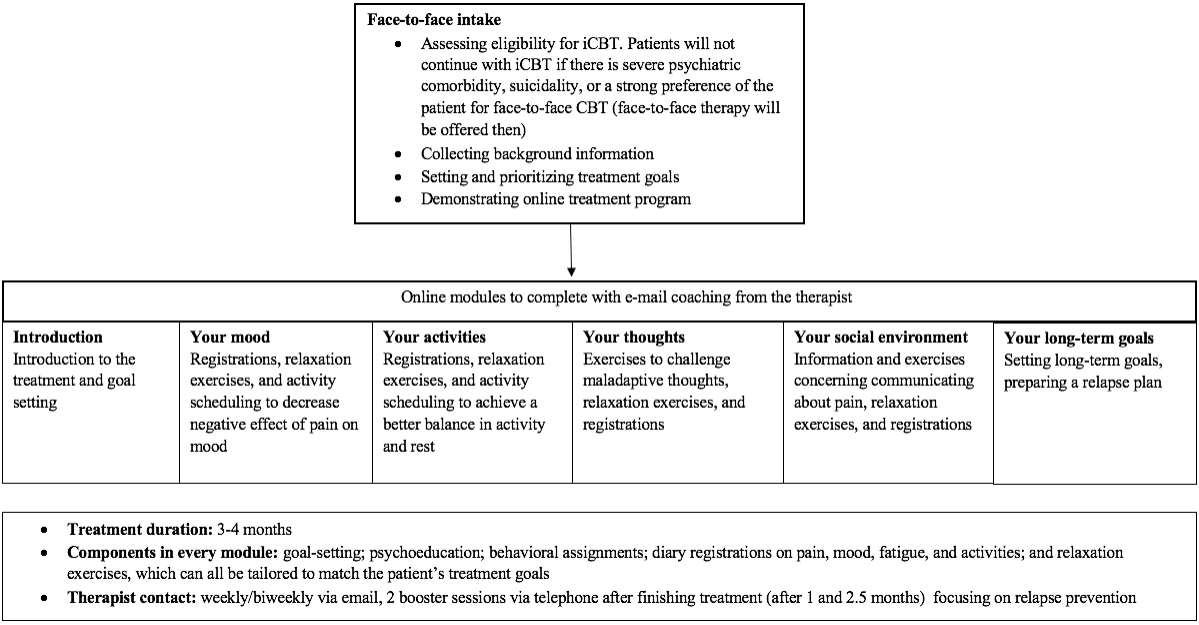
Content of the internet-based cognitive behavioral therapy (iCBT) intervention Master Your Pain.

**Figure 2:**
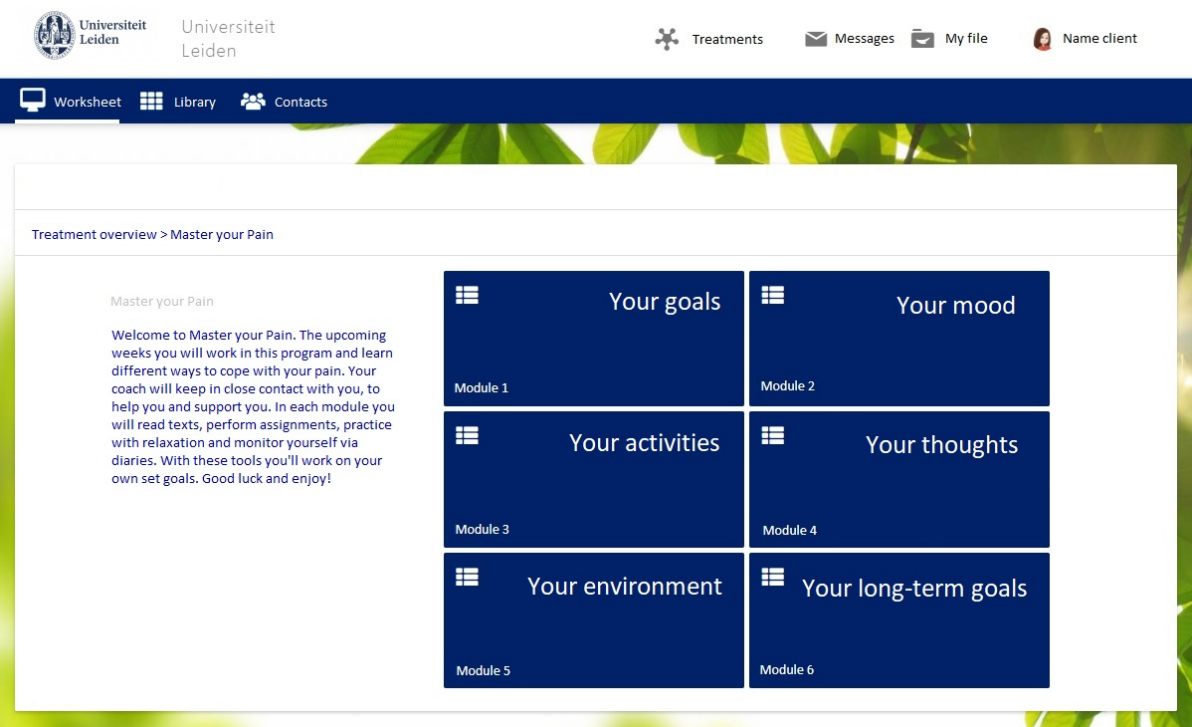
Master Your Pain homepage.

A mixed methods design was used in which a qualitative observational study was performed to test the usability of a Dutch iCBT called Master Your Pain for people with chronic pain (see Figure 1 for the content of the program and Figure 2 for a screenshot of the interface) [14], and a quantitative questionnaire was used to collect data on usability-related variables. Master Your Pain is derived from an evidence-based iCBT platform for chronic somatic conditions [15,16]. From the available treatment modules in the original platform, six modules (Your goals, Your mood, Your activities, Your thoughts, Your social environment, and Your long-term goals) were created to comprise an iCBT focused on coping with chronic pain using psychoeducation, assignments, diary registrations, and relaxation exercises. Communication with the therapist takes place via secure email messages.

### Participants

To establish a representative sample, participants were recruited in two different mental health care institutions from two geographically distinct regions in the Netherlands. One institution specializes in treating patients with fibromyalgia and the other in treatment of medically unexplained symptoms and patients of older age with psychiatric problems. The inclusion criteria were being aged 18 years or above, having some internet experience (assessed by asking about home access to the internet and experience with common online activities such as emailing and online banking), having experience with a Word operating system, and mastery of reading and writing in the Dutch language. Health care providers from the institutions informed all their patients within a certain time frame (March and April 2016 and from June to December 2017, respectively) about the study by offering an information letter regarding the goal of the study, its procedure, location, estimated time investment, compensation, and participants’ rights during the study. If patients were interested, care providers would check the inclusion criteria. Upon eligibilty determination, the researcher was contacted by email to invite the patient for the study. Before the start of the study, participants were asked to sign an informed consent form. This informed consent form contained information about the permission to record video and audio material, anonymity of the participants, confidential use of data, and the possibility to stop at any time without having to state any reasons. After three consecutive assessments without new usability problems occurring, data saturation was reached. A total of 34 participants took part in the study. However, one participant appeared to have experience with the iCBT and one was incorrectly included, since this person never used the internet (and was not able to). Both were excluded from the analyses.

### Procedure and Materials

The procedure took 60 to 90 minutes per participant. Participants were first asked to complete a questionnaire. The questionnaire started with items regarding demographic characteristics (age, sex, and educational level) and internet use (Through which medium do you have access to the internet? How often do you use the internet? Have you used the internet before to look up health-related information? Have you ever used an online self-help tool?). Digital health literacy skills were then measured using the Digital Health Literacy Instrument (DHLI) [17]. This 21-item questionnaire assesses 7 digital health literacy skill categories: operational skills, navigation skills, searching for information, evaluating information, applying information, adding content to the Web, and ensuring privacy. Questions are answered on a 4-point response scale, ranging from very easy to very difficult or from never to often. A mean score (ranging between 1 and 4) can be calculated if at least 18 items are completed. When calculating the mean scores, item scores are reversed, so that a higher score represents a higher level of digital health literacy. This instrument has been validated in a general sample of the Dutch population, with a mean of 3.11 (SD 0.45), Cronbach alpha=.87. Content and construct validity showed to be adequate [17].

After finishing the questionnaire, participants were asked to take place behind a PC (using Windows 10). They were asked to perform 8 tasks in the Master Your Pain program using a mock account created for the study. The tasks were set up as representative as possible, reflecting actual use during treatment and including all kinds of different functionalities of the intervention. The performance tasks were based on the health-related internet skills found by Van der Vaart et al [12]. All tasks required more than one skill, but they were designed so that one skill was predominantly required for each task: logging in (operating the computer and internet browser), navigating forward and back to the homepage (navigating and orienting), opening a specific assignment (using search strategies), performing an assignment (evaluating relevance), sending a message to the therapist (adding personal content to the Web), and logging out from the website (protecting and respecting privacy). One skill, evaluating reliability, was not explicitly tested since this skill is very relevant using the World Wide Web but is less relevant on a treatment platform provided by a university. The exact tasks and associated skills can be found in Multimedia Appendix 1. To prevent learning effects, performance tasks 3 to 7 were presented in random order. Tasks 1, 2, and 8 appeared in the same order for everyone, since they represented logging in (task 1), getting familiar with the program (task 2), and logging out (task 8).

Prior to the performance tasks, the researcher explained the instructions and basic information about the iCBT to the participants. It was emphasized that the focus of the study was on usability testing rather than on measuring performance in order to reduce any performance anxiety. During the tasks, participants were asked to vocalize their thoughts by thinking aloud [18,19]. This is a verbal report method that provides insight to understanding a person’s decision-making and problem-solving process. If participants stopped talking during the tasks, the researcher provided the following instruction: “Please, keep talking.” [18]. When participants had trouble finishing an assignment, a protocol was in place to allow the researcher to provide hints on how to continue. In the case a participant claimed he was not able to complete a certain task and was going to give up or when the researcher observed signs that the participant was not able to continue for whatever reason, the researcher asked, “Would you stop at this point if you were using the application on your own?” If the answer was yes, the researcher asked, “May I give you a hint in order to continue?” If the answer was yes, the researcher gave a hint. FlashBack Express screen recorder (Blueberry Software) was used to record screen activity including webcam video and audio.

### Data Analysis

Analyses were performed using the SPSS Statistics 23 (IBM Corp). Descriptive analyses were used to describe the sample’s demographic characteristics and actual internet use. Mean scores and Cronbach alpha were calculated for the DHLI. The video files with screen activity and webcam recordings were used to derive two task variables. First, the level of completion per task was rated as not completed (score 0), completed with help (score 1 when a hint was provided), or completed independently (score 2), and then summed with a possible range from 0 to 16. Second, all encountered problems per task were registered using qualitative inductive analysis [20] and classified into the 6 health-related internet skills [12]. This way, an overview was created of the types of problems that occurred regarding each type of internet skill. Two researchers at each institution coded all tasks for every participant independently, using the same coding scheme. Differences in coding were discussed until consensus was reached. Subsequently, researchers pooled and labeled similar problems. Categories that consisted of a single occurred problem were excluded, because their relevance was deemed too small. Once the assessed performance task variables were agreed upon, they were entered in SPSS, and the total number of encountered problems was calculated for each participant. Normality was tested with a Shapiro-Wilk test for all variables, considering the small sample size. For the second aim of the study, Spearman ρ correlations were calculated between the two outcome measures task completion and number of problems encountered and the three variables that were assumed to be associated: age, educational level, and DHLI scores.

## Results

### Participants

The sample of 32 participants (Table 1) was 84% women (27/32), and the sample’s age range was 22 to 75 years (mean 49.9 [SD 15.69] years). The majority had a high school or vocational school education level (13/32, 41%) and lived with a partner, kids, or parents (20/32, 63%). A laptop was the main device (29/32, 91%) used to access the internet at home; the other 3 participants had internet access at home via a PC. Internet was used (almost) every day by most participants (26/32, 81%). Of the participants, 97% (31/32) had searched for health-related information on the internet before. None of the participants ever used an online health intervention (data not shown). The average score on the DHLI was 2.90 (SD 0.48) on a scale from 1 to 4. Minimum and maximum scores ranged from 1.21 to 3.62, and Cronbach alpha=.86.

**Table 1:** Sociodemographics and internet use (N=32).

Characteristics		Value
**Gender, n (%)**		
	Men	5 (16)
	Women	27 (84)
Age (years), mean (SD)		49.9 (16)
**Living situation, n (%)**		
	Alone	12 (38)
	With partner	11 (34)
	With partner and kids	6 (19)
	With parents	3 (9)
**Educational level, n (%)**		
	Primary school	12 (38)
	High school or vocation	13 (41)
	College degree or higher	7 (22)
**Internet access^a^, n (%)**		
	Laptop	29 (91)
	Smartphone	17 (53)
	PC	6 (19)
**Frequency of internet use, n (%)**		
	(Almost) every day	26 (81)
	Multiple days a week	3 (9)
	Once a week or less	3 (9)
Digital health literacy^b^, mean (SD)		2.90 (0.48)

^a^Respondents could mark more than one answer on this item.

^b^Measured by the Digital Health Literacy Instrument.

### Completion of the Performance Tasks

All performance tasks except one could be completed independently by more than 50% of the participants (Table 2). Task 7 (fill in a diary registration) was most often not completed, and participants most often needed help with performance task 1 (log in to the platform). Tasks 1, 4, and 6 could not be completed by a participant during testing (different participants).

**Table 2:** Completion and performance per performance task (N=32).

#	Assignment	Completed independently, n (%)	Completed with help, n (%)	Not completed, n (%)
1	Log in to platform^a^	15 (47)	12 (38)	1 (3)
2	Navigate to assignment and back to homepage	22 (69)	5 (16)	5 (16)
3	Send message via mailbox in platform	22 (69)	7 (22)	3 (9)
4	Search assignment	27 (84)	4 (13)	1 (3)
5	Perform an iCBT^b^ assignment	24 (75)	5 (16)	3 (9)
6	Read a text and recall the core message	29 (91)	2 (6)	1 (3)
7	Fill in a diary registration	14 (44)	7 (22)	11 (34)
8	Log out of platform	18 (56)	6 (19)	8 (25)

^a^n=29 (due to a program error during start-up, assignment 1 could not be completed as requested by 4 participants; the research leader helped them to get onto the platform).

^b^iCBT: internet-based cognitive behavioral therapy.

### Usability Problems Encountered in the Performance Tasks

A summary of usability problems is provided in Table 3. Usability problems occurred on all skill levels, some being more general (eg, regarding operating the computer and internet browser) and others being more specific to the iCBT (eg, regarding navigating and orientation skills to find a specific button or assignment).

Concerning operating the computer and internet browser, problems using the keyboard were observed in each performance task in which the keyboard was needed (12/32), and problems with general browser button knowledge were also relatively common (15/32). These operational problems ranged from typing errors to not knowing how to use the backspace or using the address bar incorrectly (eg, participants entered their log-in data instead of the Web address of the treatment program in the address bar). In navigating and orientation skills, problems with locating or properly using buttons on the iCBT platform were most common (such as returning to the homepage in task 2, finding the email box in task 3, finding the diary button in task 7, or logging out in task 8; 24/32). Concerning using search strategies, two main problems were observed related to selecting the right module and the right assignment (task 2). Selecting an incorrect assignment (choosing an irrelevant assignment or choosing an assignment participants were not supposed to select) was the most prevalent problem (15/32). Finding the diary registration (task 7) appeared to be especially problematic (14/32). In evaluating relevance and reliability, problems occurred in understanding the meaning of the text. For example, many participants were not able to understand and recall the core message of a text (task 6, 11/32). In the skill adding personal content to the Web, problems occurred writing a text or message as part of an exercise or in communication with the therapist (task 3 and 5). This could be related to writing skills (eg, using punctuation; 16/32), formulating a message (eg, incorporating all relevant information; 10/32), or using the appropriate fields for each part of the information (eg, distinguishing recipient and subject fields in task 3; 11/32). Concerning skill protecting and respecting privacy, one specific problem was experienced by more than half of all participants: neglecting to click on the close button in the browser to protect the action when asked to log out of the iCBT platform in task 8 (18/32).

**Table 3:** Observed problems per performance task and number of participants encountering these problems (N=32). Participants could have encountered a problem multiple times, so the rows are not cumulative.

Assignment		1. Log in, n (%)	2. Navigate, n (%)	3. Write, n (%)	4. Search, n (%)	5. Perform, n (%)	6. Recall, n (%)	7. Use diary, n (%)	8. Log out, n (%)
**Problems with operational skills**									
	Limited general browser button knowledge	15 (47)	—^a^	2 (6)	—	—	—	4 (13)	
	Limited general keyboard knowledge	8 (25)	—	2 (6)	—	3 (9)	—	1 (3)	
	Typing errors	7 (21)	—	—	—	1 (3)	—	—	
	Inadequate slider use		—	—	—	—	—	7 (22)	
	Difficulty reading from monitor	3 (9)	—	—	—	1 (3)	1 (3)	—	
**Problems with navigation skills**									
	Locating/proper use of buttons on platform	—	9 (28)	16 (50)	—	—	—	4 (13)	13 (41)
	Not recognizing homepage as such	—	5 (16)	—	—	—	—	—	—
**Problems using search strategies**									
	Selecting incorrect assignment/not finding correct assignment	—	4 (13)	—	2 (6)	—	—	14 (44)	—
	Selecting incorrect module	—	1 (3)	—	2 (6)	—	—	—	—
**Problems evaluating relevance**									
	Not understanding core message/goal of assignment	—	—	—	—	8 (25)	11 (34)	—	—
**Problems adding content**									
	Language error or incorrect spelling	—	—	16 (50)	—	—	—	—	—
	Uncommon use of email etiquette	—	—	14 (44)	—	—	—	—	—
	Incorrect use of text fields	—	—	4 (13)	—	7 (22)	—	—	—
	Providing incomplete information	—	—	10 (31)	—	—	—	—	—
**Problems ensuring privacy**									
	Unprotected log-out	—	—	—	—	—	—	—	18 (56)

^a^Not applicable.

### Correlations Between Usability Variables and Age, Educational Level, and Digital Health Literacy

Table 4 shows the correlations between the number of completed performance tasks and number of encountered problems with age, educational level, and the sum scores on the DHLI, respectively. Age showed to be moderately related to both dependent variables with significant correlations of ρ=–.64 and ρ=.65, respectively, meaning that older age was associated with a lower number of completed tasks and a higher number of encountered problems. Scores on the DHLI were moderately correlated with the number of completed tasks (ρ=.46), meaning that higher scores on this instrument were related to a higher number of completed tasks. Scores on the DHLI did not correlate with the number of encountered problems, and educational level did not show any significant correlations either. Age, educational level, and scores on the DHLI did not correlate with each other (not shown).

**Table 4:** Spearman ρ correlations between usability variables and age, educational level, and digital health literacy (N=32).

Completion and encountered problems	Age ρ	*P* value	Educational level ρ	*P* value	DHLI^a^ ρ	*P* value
Number of completed performance tasks	–.638	<.001	.292	.13	.456	.02
Number of encountered problems	.645	<.001	–.103	.58	–.114	.54

^a^DHLI: Digital Health Literacy Instrument.

## Discussion

### Principal Findings

In this study, we investigated the usability of an iCBT and its relationship with age, educational level, and digital health literacy of potential users. This provided insight into both what needs to be improved in our intervention and what type of people will need extra assistance in using it.

By observing the rate at which the assignments in the study could be completed and the type of problems that occurred, several conclusions can be drawn. First, some operational, navigational, and search tasks (eg, asking for the more practical digital skills) caused difficulties for many participants. Summarizing the major repeated problems, these occurred during logging in, logging out, finding the email box, and finding the diary registrations. A large proportion of the participants needed assistance to complete these tasks. Considering the high number of people who struggled with these tasks, it would be reasonable to conclude that these issues are inherent in the usability of the program. These matters should be addressed by changing button locations or making sure that these buttons stand out more.

A number of problems occurred that were more related to the practical digital skills of the participants and could not be attributed to the design of the platform. These nonplatform-related problems were rooted in either lack of general orientation competence (eg, locating keys on a keyboard or finding buttons on an internet browser) or navigation on a website (eg, understanding what a homepage is and how to navigate through its pages). For participants who encountered these type of problems, an introduction to the platform given by the therapist, other support staff, or instructional video could probably help them overcome the large majority of these struggles. Showing patients how to log in and log out, how the platform is built, and where to find all the parts of the intervention would help them in getting started. Descriptive analyses showed that many of the navigational problems were present during the first encounter with a new part of the website and were not repeated in the later assignments. This validates the conclusion that some level of support would suffice to overcome these problems (especially when combined with the suggested design alterations to the platform, which would increase the intuitiveness on the website).

Another substantial number of observed problems related to more complex digital health literacy skills such as being able to properly understand and use the content of the intervention and being able to express oneself in writing (both in terms of grammar and content). Problems in these areas are not easily overcome with basic support. This indicates that an online iCBT program would not be suitable to use as a stand-alone replacement to regular face-to-face therapy for people experiencing these struggles. The results show that a subgroup of people would not be able to grasp the key messages from the therapy and would therefore probably not be able to use it beneficially. Moreover, they would not be able to communicate this to their therapist or be able to ask for feedback or support to do so. For this group, the platform might be used as support in a regular treatment (eg, using diary registrations, relaxation exercises, or simple assignments and psychoeducational texts). A blended version of therapy with both online and face-to-face sessions could meet these needs. The face-to-face sessions should then incorporate the core elements of CBT and focus on getting the theory across by helping patients in understanding and practicing it. The online platform could offer support for the more practical parts of the treatment [7]. Additionally, to deliver more benefits of online therapy, it would be valuable to look further than the rather general approach to iCBT, which provides information and assignments in a text-based manner. The use of online tools greatly supports other means of communication, using video, voiceover, and even gaming elements. By offering more creative options, users with lower (health) literacy skills could benefit more from iCBT, and it would additionally make the interventions more attractive and pleasurable to use.

Which level of support is needed by whom remains an unanswered question. A subgroup of patients could follow the program independently, a subgroup would need practical assistance, and a subgroup would need a blended version in which the theoretical content is provided face-to-face. The data in this study support a cautious proposal for this divide. First, it is must be emphasized that for this study, only those people with internet experience who used the computer and internet on a regular basis were included. This has shown to be the first prerequisite to consider starting iCBT. Second, the associations found in this study provide a starting point for deciding in which manner the iCBT could be used independently. Although the sample size in this study was small and the analyses can therefore only be explorative, the associations can give helpful indications. Correlations show that age is a large factor to consider. Our findings show that elderly patients with internet experience are able to use the iCBT but experience significantly more struggles than younger people. This corresponds with previous studies by Van Deursen [13,21] that showed older people more often have lower operational and navigational skills in comparison with younger people. This discrepancy could be resolved by offering eligible elderly patients an introductory session to the platform to help them through the program for the first time. This could also serve the purpose of screening whether their digital skills are sufficient. Furthermore, correlations were shown between the number of completed tasks in our intervention and scores on the DHLI. This corresponds with previous research, which shows that digital health literacy is a predictor of critical internet use [22]. Nevertheless, measuring digital health literacy is a challenge due to the complexity of the concept. The DHLI, however, might be a usable screening instrument. This would foremost be relevant to check whether patients possess complex skills such as evaluating relevance of information and adding content to the Web. In this study, correlations were only shown between the DHLI and number of completed tasks, not with the number of problems that occurred. This might be explained by the usability problems attributed to the interface on the website. Many participants struggled with these problems, so the skill level of the participants is most likely not the only issue. It could be hypothesized that people with better digital health literacy (and thus a higher score on the DHLI) were able to overcome these problems better and still complete the task, while people with lower digital health literacy (a lower score on the DHLI) were not able to.

The lack of association between educational level and usability of the platform was quite remarkable. An explanation for this could be that the study sample consisted of an overrepresentation of older people. Among this subgroup, the distribution of educational level is often different, since more people had lower education levels in previous decades, not because of their capabilities but because of other practical reasons [23]. Nevertheless, in our sample, no relationship between age and educational level was found, which would indicate that educational level is simply not related to the usability of this iCBT, implying that online interventions can be feasible to use regardless of the educational level of a patient.

### Limitations

This study has a number of limitations. First of all, it should be considered that this paper describes the usability of a specific intervention, Master Your Pain, and these results cannot be generalized to all iCBT interventions. This also accounts for the relationships found between age and digital health literacy. However, we do feel that the level of usability is generally characterized by a combination of the ease of use of an online tool and the skills of those who use it. Therefore, while our results are not simply generalizable, they do provide insight into what to take into account on a broader level regarding iCBT interventions and other online (health) tools. Principles of user experience design are key to take into account in the developmental phase of any digital tool. Second, the usability study was not conducted in a naturalistic setting but in a lab with one of the researchers directly present. This may have influenced the performance of the participants. Third, this study comprised a small sample. For a usability test, this sample was actually quite large, as smaller groups are more common [24]. Conducting this type of research is labor intensive and regularly qualitative in nature. However, to also conduct statistical analyses on the data, as was done in this study, a larger number of participants is needed to draw firm conclusions. Also, from technology-design theory, it is advised to perform a usability study in several rounds, with necessary design alterations in between rounds in order to evaluate if previous operational and navigational usability issues were overcome [24]. This could have helped in accurately determining which problems were related to the design of the platform and which problems were related to the digital health literacy skills of the participants. Finally, the sample that was used is not free of bias, since only people with some internet experience were included; it could also be suggested that only people with an interest in online therapy would consider joining this type of study. Nevertheless, the sample was rather representative for eligible users of a chronic pain iCBT, since these patients are generally somewhat older and females are overrepresented. Moreover, the recruitment was done in two very different geographical regions in the Netherlands. Also, people without any internet experience or interest would not be suitable for iCBT in general, so it seems appropriate not to include them in this study.

### Conclusion

All in all, when developing a new internet intervention it is key to test its usability on all domains of the digital health literacy spectrum, including the intended target group. Our study strongly supports this notion and asks for specific attention for older individuals and people with a low level of health literacy. In addition to the practical design alterations, such a test will provide insight into the level of digital health literacy needed to benefit from the program and provide indications on what type of support is needed by whom. Regarding our own interventions, we continuously strive to improve upon them and we will solve the easy to adjust usability issues in the next release of Master Your Pain. The issues regarding skills of users will be addressed in our therapist training so that therapists are keen on offering new patients a user experience that fits their needs and skills.

## Appendix

Multimedia Appendix 1 Usability assignments.

## Abbreviations

DHLI: Digital Health Literacy Instrument
iCBT: internet-based cognitive behavioral therapy
