# Supplementary material for: The Role of Age, Education, and Digital Health Literacy in the Usability of Internet-Based Cognitive Behavioral Therapy for Chronic Pain: Mixed Methods Study
Source: JMIR Form Res. 2019 Nov 21;3(4):e12883. doi: 10.2196/12883 (PMC6914283; doi:10.2196/12883)
Supplement: Multimedia Appendix 1 [file formative_v3i4e12883_app1.docx]

Multimedia Appendix 1. Usability assignments.

**Assignment 1 [operating the computer and Internet browser]**

Imagine this is the first time you are using online therapy. You are at home, behind your PC and you have opened your internet browser.

1. Go to this website [URL master your pain – uleiden.karify.com]
2. Log in with the following account: [user name and pass word are provided]
3. You have entered the homepage of “Master your pain”. You can take a moment to look at the homepage, without clicking any buttons

**Assignment 2 [Navigating and orientating]**
Your therapist has asked you to do an assignment in module ‘ Your mood’ , in order to help you relax.

1. Navigate to the module ‘Your mood’
2. Click on the ‘Relaxation exercise’
3. Scroll through the assignment (you don’t have to read the complete text), until you are at the end of the assignment
4. Go back to the Homepage.

**Assignment 3 [Adding personal content to the web]**

Your therapist asked you to do the ‘Energy points’ assignment. However, your pain is so severe at the moment that you are not able to do this. You want to inform your therapist about this and reschedule the deadline for completing the assignment.

1. Go to ‘My messages’ and open a new message
2. Choose ‘Test Therapist’ as recipient
3. Choose a topic for you message
4. Explain you situation and question to your therapist
5. Send the message
6. Go back to the Homepage.

**Assignment 4 [Utilizing search strategies]**
You receive little support from your colleagues at work and you are struggling with asking them to help you more often. ‘Master your pain’ contains an assignment regarding asking support. In deliberation with your therapist you decided to do this assignment.

1. Find this assignment
2. Call out the name of the assignment. You do not have to open it or read it.
3. Go back to the Homepage.

**Assignment 5 [Evaluating relevance and reliability of web content]**

Your daily activities can influence the pain you perceive. Imagine that your therapist has asked you to perform an assignment to monitor your activities during a few days, go gain insight into your rest-activity balance.

1. Go to the module ‘Your activities’
2. Open the assignment ‘Energy points’
3. Read the information on the assignment and complete the assignment by filling in the questions on page 2.
4. Go back to the Homepage

**Assignment 6 [Evaluating relevance and reliability of web content]**

Your therapist has asked you to start the module ‘Your activities’

1. Go to the module ‘Your activities’
2. Open assignment ‘Introduction’
3. Read the first page of the assignment
4. Formulate the core message of this text in one sentence. Please state this message aloud.
5. Go back to the Homepage

**Assignment 7 [Adding personal content to the web]**

In ‘Master your pain’ you are supposed to fill out a diary each day.

1. Go to the module ‘Your mood’ and open the ‘Mood diary’
2. Read the assignment and fill out the diary
3. Send the diary to your therapist.
4. Go back to the Homepage.

**Assignment 8 [Protecting and respecting privacy]**

You want to log out of the website

1. Log out
